# Feasibility Study on the Design and Synthesis of Functional Porous Organic Polymers with Tunable Pore Structure as Metallocene Catalyst Supports

**DOI:** 10.3390/polym10090944

**Published:** 2018-08-24

**Authors:** Xiong Wang, Cuiling Zhang, Wenxia Liu, Pingsheng Zhang

**Affiliations:** Lanzhou Petrochemical Research Center, Petrochemical Research Institute, PetroChina, Lanzhou 730060, China; zhangcuiling3@petrochina.com.cn (C.Z.); liuwenxia1@petrochina.com.cn (W.L.); zhangpingsheng@petrochina.com.cn (P.Z.)

**Keywords:** porous organic polymer (POP), metallocene catalyst, ethylene polymerization, pore structure

## Abstract

Porous organic polymers (POPs) are highly versatile materials that find applications in adsorption, separation, and catalysis. Herein, a feasibility study on the design and synthesis of POP supports with a tunable pore structure and high ethylene-polymerization activity was conducted by the selection of functional comonomers and template agents, and control of cross-linking degree of their frameworks. Functionalized POPs with a tunable pore structure were designed and synthesized by a dispersion polymerization strategy. The functional comonomers incorporated in the poly(divinylbenzene) (PDVB)-based matrix played a significant role in the porous structure and particle morphology of the prepared polymers, and a specific surface area (SSA) of 10–450 m^2^/g, pore volume (PV) of 0.05–0.5 cm^3^/g, bulk density with a range of 0.02–0.40 g/cm^3^ were obtained by the varied functional comonomers. Besides the important factors of thermodynamic compatibility of the selected solvent system, other factors that could be used to tune the pore structure and morphology of the POP particles have been also investigated. The Fe_3_O_4_ nanoaggregates as a template agent could help improve the porous structure and bulk density of the prepared POPs, and the highly cross-linking networks can dramatically increase the porous fabric of the prepared POPs. As for the immobilized metallocene catalysts, the pore structure of the prepared POPs had a significant influence on the loading amount of the Zr and Al of the active sites, and the typically highly porous structure of the POPs would contribute the immobilization of the active species. High ethylene-polymerization activity of 8033 kg PE/mol Zr h bar was achieved on the POPs-supported catalysts, especially when high Al/Zr ratios on the catalysts were obtained. The performance of the immobilized metallocene catalysts was highly related to the pore structure and functional group on the POP frameworks.

## 1. Introduction

Porous organic polymers (POPs) have received a staggering degree of attention in various research areas, including adsorption, separation, and heterogeneous catalysis, owing to their huge surface area, tunable pore size, flexible synthetic strategy, and readily modifiable functionality [1,2,3,4,5,6,7,8,9]. Numerous studies on these porous organic materials, including covalent organic frameworks (COFs) and porous coordination polymers (PCPs) or metal-organic frameworks (MOFs), as olefin polymerization-catalyst supports and mobilization procedures have been investigated [10,11,12]. Unlike inorganic supports, such as silica gel, zeolites, molecular sieves, which need complex chemical treatments to get rid of acidic groups on their surfaces and present residual inorganic fragments within the produced polyolefins that may affect their mechanical and optical properties [13,14,15,16], POPs offer significant advantages over their inorganic equivalents: they provide a much closer analogue to the environment prevailing in the homogeneous polymerization, do not require a fastidious immobilization procedure, and should not significantly affect the final polyolefin properties [17,18,19,20].

The rich variety of organic building blocks combined with the diverse polymerizations has led to various types of novel POPs [21,22,23,24,25], and POPs could be divided into two categories, amorphous and crystalline, according to the crystalline tendency of the molecular chains of the polymers. Generally, the pore structure and topologies of the crystalline POPs are well-defined, including crystalline covalent organic frameworks (COFs) and porous coordination polymers (PCPs) or metal-organic frameworks (MOFs) [26,27,28]. The absence of functional groups such as catalytic sites, however, renders them relatively nonspecific, as porous materials for applications such as heterogeneous catalysis and others, and the targeted functional groups should be incorporated in the organic frameworks in order to accommodate active sites [21]. Pre- and postsynthetic modification methods were developed to functionalize the crystalline POPs. Unfortunately, the routes and methods of preparation of immobilized olefin polymerization catalysts are usually complex and the polymerization activities are unsatisfactory compared to other industrial catalysts [29,30]. Furthermore, the incorporation of functional groups and active sites, in turn, would cause defects on the frameworks broadening the pore-size distribution of the pore structure [21,31].

Amorphous POPs including polymers of intrinsic microporosity (PIMs), highly cross-linked networks (also called microporous polymer networks, MPNs), and conjugated microporous polymers (CMPs) are formed under kinetic control and show no long-range molecular order [32,33,34]. Typically, they have a wide range of pore-size distribution and unspecified topologies. The synthetic strategy and the post modifications of this kind of POPs, however, are pretty flexible due to diverse synthetic methods and suitable functional monomers to incorporate the active species of olefin polymerization. The produced olefin catalysts immobilized on this kind of POPs are typically highly active in terms of olefin (co)polymerization. In our previous work [35,36,37], we have reported that the pore size and pore-size distribution of the produced HEMA-functionalized POPs are highly tunable by a dispersion polymerization strategy, and the ethylene polymerization and copolymerization activities of the produced POPs-supported metallocene catalysts are higher than their conventional silica gel-supported counterparts; furthermore, the molecular chain structure of the polyolefin could be tailored due to the confinement effect of the nanopores of the POPs-supported catalysts during the process of the olefin polymerization. Therefore, it would be beneficial to design and prepare porous organic polymers with a controlled pore structure and functional groups to incorporate the active sites for olefin polymerization-catalyst supports.

In this work, we have investigated the design and preparation of 2-hydroxypropylmethacrylate (HPMA), glycidyl methacrylate (GMA), vinylbenzyl chloride (VBC), and dual-functional comonomer-functionalized POPs with a controllable pore structure, surface morphology, and bulk densities through a dispersion polymerization strategy. The POPs-supported metallocene catalysts showed excellent ethylene-polymerization activities, and the pore structure of the POPs had significant influence on polymerization activity.

## 2. Materials and Methods

### 2.1. Materials

GMA, HPMA, and 2-hydroxyethylmethacrylate (HEMA) (≥98%) were passed through an oxide aluminum column (neutral) to remove the inhibitor before use. Divinylbenzene (80%/55%, mixtures of isomers) and styrene (≥98%) were treated with an NaOH solution (10% weight) to remove the inhibitor, and were washed with distilled water until neutralization. VBC (≥97%) was passed through an active oxide aluminum column before use. 2,2′-Azo-bis-isobutyronitrile (AIBN) (≥99%), poly(vinyl alcohol) (PVA, 1788), iron oxide(II,III) (Fe_3_O_4_) nanopowder (99.5%, 20 nm beads), and ethanol (≥99.7%) were used without further purification. All these above agents were purchased from Aladinn Reagent, Shanghai, China. Bis (n-butylcyclopentadienyl) zirconium dichloride ((n-BuCp)_2_ZrCl_2_) (≥98%) was purchased from DAL CHEM (Nizhny Novgorod, Russia) and methylaluminoxane (MAO) (10% toluene solution) was provided from PetroChina, Lanzhou, China and they were used directly as received.

### 2.2. Preparation of the Functionalized POP Supports

Functionalized POP particles were synthesized according to a dispersion polymerization or precipitation polymerization method by previous references [12,35]. Briefly, 130 mL solvent was charged into a 250 mL glass reactor, then 5.0 g (0.0384 mol) of divinylbenzene (DVB) (80%) and 0.0256 mol functional monomer(s) (HEMA, HPMA, GMA, or VBC) and 2 wt % of PVA were added into the reactor when stirring. After the monomers and the stabilizer were dissolved in the solution, 2 wt % of the initiator AIBN was added into the reactor to initiate the free-radical polymerization at 70 °C for 1–4 h. After aging for 3–6 h at 80 °C, the product was purified by ethanol/distilled water-washing and vacuum-dried for further use. When Fe_3_O_4_ nanopowder was used as a template to prepare the target product, the similar procedure was adopted except that 3 wt % Fe_3_O_4_ nanoaggregates were added into the reactor before the addition of the initiator AIBN, and that the prepared particles were washed twice with hydrochloric or sulfuric acid for 1–2 h to remove the template before the products were washed and vacuum-dried to remove impurities.

### 2.3. Catalyst Immobilization

The single-site catalyst was immobilized on the prepared functionalized POPs after they were vacuum-dried. All supporting procedure was free from air and moisture under high-purity nitrogen according to a previous work [35]. Typically, in a 250 mL glass reactor, 3.0 g treated functionalized POP particles and 80 mL toluene, and a 20 mL MAO solution were charged into the reactor and stirred at ambient temperature for 1–2 h. Then, 0.30 mmol (n-BuCp)_2_ZrCl_2_ was added to the MAO-treated POPs, and the suspension was kept stirring at –20 °C for 2–3 h. Then, the obtained solid was washed and vacuum-dried to get the final supported metallocene catalyst.

### 2.4. Characterization

Nitrogen sorption was analyzed on a Nova 2000e (Quantachrome Instruments, Boynton Beach, FL, USA) at liquid nitrogen (77.3 K). The POP samples were tested in a glass tube before the samples were vacuum-dried at 120 °C for 12 h to remove adsorbed materials, and the supported catalysts were directly tested under N_2_ protection without heating for desorption. Al and Zr loading of the supported catalysts were performed on a VISTA ICP-MPX (VARIAN, Palo Alto, CA, USA). 0.1 g catalyst was dissolved in a 10 mL aqua regia by heating, and then the solution was metered to 100 mL constant volume using 2% HCl solution. Al and Zr loading contents were obtained by the standard curves of their characteristic peaks. IR analysis was performed on a NEXUS 670 FTIR (Glendale, WI, USA). The surface morphology of the POP samples was conducted on a scanning electron microscope (SEM, Philips, XL20, Amsterdam, The Netherlands). The functionalized POP samples were mounted on electric glue, then sprayed with a thin layer of gold in vacuum before testing.

## 3. Results and Discussion

### 3.1. Preparation of Poly(divinylbenzene) (PDVB)-Based Functionalized Porous Organic Polymers

In this work, the emphasis focus on the design and synthesis of potential DVB-based porous organic polymer supports with different functional comonomers for metallocene catalysts by a dispersion polymerization method [12,35], and the suitable polymer supports, like the inorganic counterparts, generally contain relatively high specific surface area (SSA) and porosity (SSA ≥ 100 m^2^·g^−1^, PV ≥ 0.2 cm^3^·g^−1^) with good morphology and suitable bulk density (0.2–0.4 g·cm^−3^). A series of functionalized PDVB-based POP particles were prepared using St, HEMA, HPMA, GMA, and VBC as comonomers.

**Figure 1:**
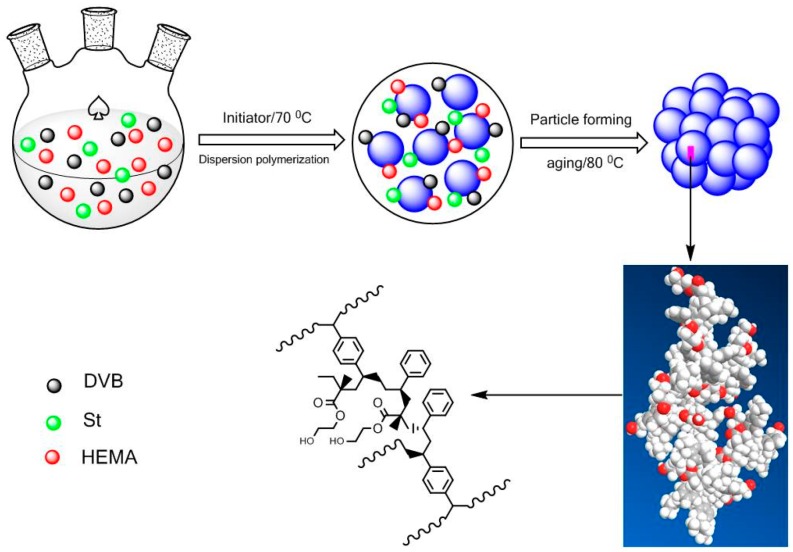
Schematic illustration of synthesis of functionalized PDVB particles.

The schematic illustration of synthesis of functionalized PDVB particles is shown in Figure 1. The porosity results based on N_2_ sorption results and bulk density are listed in Table 1. Non Local Density Functional Theory (NLDFT or DFT) simulation was adopted using N_2_-carbon kernel at 77 K based on a slit-pore model to evaluate the pore textural structure, especially the pore-size distribution of the prepared POP samples.

### 3.2. The Influence of Functional Comonomers on the Pore Structure of POPs

**Figure 2:**
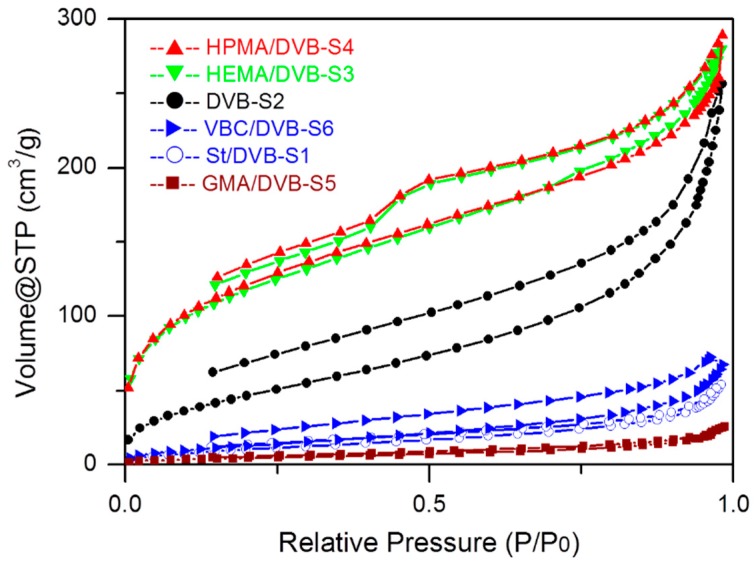
N_2_ sorption isotherms of porous organic polymers synthesized with different functional comonomers.

The influence of functional comonomers on the formation of a nanoporous structure was investigated firstly. As shown in Figure 2, quite different N_2_ sorption isotherms (Samples 1, 3, 4, 5, and 6 with St, HEMA, HPMA, GMA, VBC as functional comonomers, respectively, and Sample 2: DVB only) mean that quite different porous structure were produced by dispersion polymerization in ethanol/deionized water (V:V = 9:1) from these different functional comonomers. The PDVB-based POPs prepared with functional comonomers such as HEMA and HPMA are highly porous, and the PDVB without other functional comonomers is moderately porous, while the POPs synthesized with GMA, VBC, and St (actually with no other functional group and non-cross-linking ability compared with DVB) are poorly porous. Therefore, the type of the chosen functional comonomer in the synthesis is an important factor of tuning the pore structure of prepared POPs. From the nitrogen sorption isotherms of these functional POPs, we can also reasonably infer that two major factors of the used comonomers could influence the porous structure of the prepared POPs. The first one is the functional group on the selected comonomer; the polarity of the prepared POPs were changed when changing the functional comonomer, which would vary the thermal compatibility between the prepared POPs with the solvent system, and the porous structure could be adjusted by the thermodynamic compatibility. Generally, a highly porous structure was produced from a thermodynamic compatible system, and a nonporous structure was generated from systems with bad thermodynamic compatibility, which could be explained by the classical pore-formation mechanism of porous polymer microspheres. The second factor is the cross-linking content in the prepared functional POPs; when a kind of functional comonomer with only one polymerizable double bond is added, the cross-linking degree is actually decrease.

**Figure 3:**
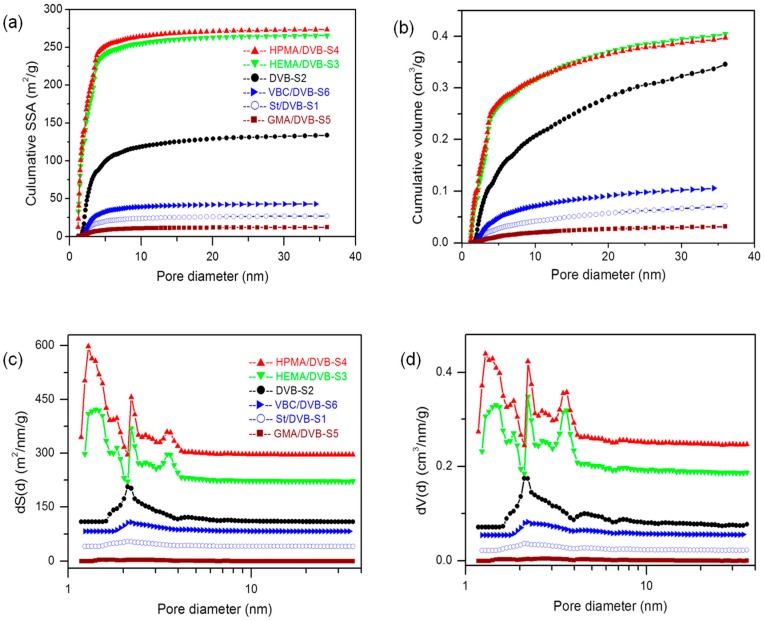
Non Local Density Functional Theory (NLDFT) simulation of pore structure of different functionalized PDVB-based POPs. (**a**) Cumulative specific surface area (SSA) curves, (**b**) porosity (PV) curves, (**c**) pore-size-distribution curves of dS(d) vs. d and (**d**) dV(d) vs. d.

Due to the amorphous nature of the prepared functional POPs, which make it impossible to characterize by X-ray crystallography, an NLDFT simulation was used to evaluate the pore size and pore-size distribution of these POPs. From Figure 3, we can see that the pore-size abundance of these POPs mainly focuses on a relatively wide range from 1 to 10 nm, except that GMA and styrene as comonomers resulted in nearly nonporous materials. HPMA and HEMA-functionalized PDVB are highly porous with well-defined cavities mainly scattering in micropores and narrow mesopores from 1.1 to 4.5 nm, and the cumulative SSA and PV of the HPMA- and HEMA-functionalized PDVB are about 250 m^2^/g and 0.264 cm^3^/g, and 240 m^2^/g and 0.256 cm^3^/g, respectively. The mode value (the highest peak) of pore diameter from the NLDFT analysis of the HPMA-functionalized PDVB is 1.3 nm in the range of micropores in the SSA and PV pore-size-distribution curves, and the mode values of pore diameter of HEMA-functionalized PDVB are 1.4 nm in the pore-size distribution of SSA and 2.2 nm in the pore-size distribution of PV in the range of micropores and narrow mesopores. Furthermore, we can see the larger pore size from above 4.5 nm contributes more to total pore volume than surface area. In contrast, the mode value of pore diameter of the VBC-functionalized PDVB is about 2.1 nm, which is similar to that of the PDVB, and the similar pore-size distribution could be explained by their approximate solubility parameters, and the decrease of abundance of pore-size distribution, despite similar pore-size distribution, could be explained by the worse thermal compatibility of VBC with the mixture solvent and the decrease of cross-linking degree by adding the non-cross-linking functional comonomer VBC.

As shown in Table 1, HEMA and HPMA are good functional comonomers to prepare porous polymers for catalyst support, with the highly porous structure of Sample 3 (SSA = 417 m^2^/g, PV = 0.434 cm^3^/g) and Sample 4 (SSA = 430 m^2^/g, PV = 0.447 cm^3^/g), while VBC, St, and GMA are typically bad for synthesis of porous polymers, at least in this mixture solvent, and they also cause low bulk density (<0.1 g/cm^3^) due to their poor thermal compatibility with the dispersion solvent. From the above pore-size-distribution curves, we can also reasonably conclude that the increase of average pore diameter from about 4 nm to 8 nm of poorly porous polymers is caused mainly by the decrease of abundance of pore-size distribution in the range of micropores and narrow mesopores.

### 3.3. The Synthesis of PDVB-Based POPs with Dual Functional Comonomers

**Figure 4:**
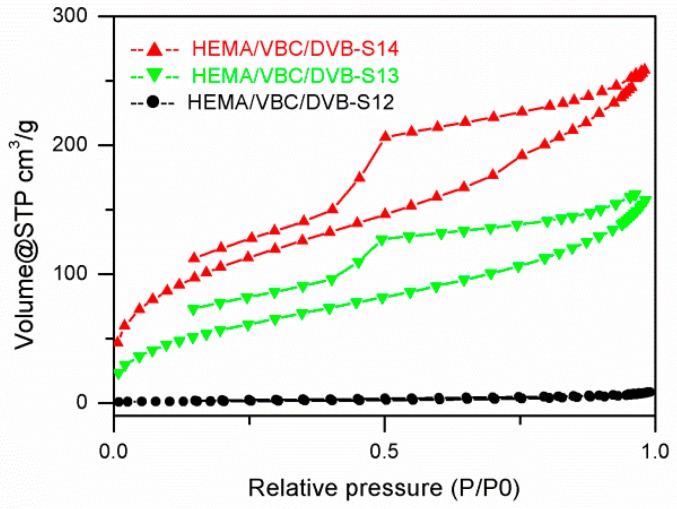
N_2_ sorption isotherms of POPs synthesized with different HEMA/VBC/DVB molar ratio.

Two functional comonomers, HEMA and VBC (or styrene), with different thermal compatibility with the dispersion solvent were used to tune the pore structure, bulk density, and particle morphology. The pore structure of the prepared PDVB-based POPs with dual functional comonomers could be controllable by tuning the HEMA/VBC/DVB molar ratio. Three samples of POPs (Samples 12, 13, and 14) were synthesized in different HEMA/VBC/DVB molar ratios, and three types of nitrogen isotherms were obtained as seen in Figure 4. When the functional comonomer VBC was in a relatively high molar ratio, the comonomer systems are inhomogeneous in ethanol dispersion solvent due to the bad solubility of VBC, so Sample 12 wasn’t synthesized in dispersion polymerization, but in suspension polymerization, and nonporous isotherm was obtained with relatively high bulk density 0.33 g/cm^3^. By decreasing the VBC molar ratio, the HEMA/VBC/DVB systems could be homogeneously dispersed in ethanol, and porous isotherms were obtained in Samples 13 and 14.

**Figure 5:**
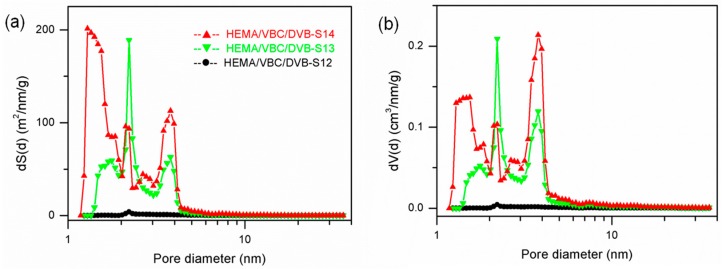
NLDFT simulation of pore structure of POPs (Samples 12/13/14) from dual functional comonomers. (**a**) Pore-size-distribution curves of dS(d) vs. d and (**b**) dV(d) vs. d.

Comparing Sample 13 with Sample 14 prepared in dispersion polymerization, we could see that the PDVB-based POPs with dual functional comonomers HEMA and VBC have a similar pore structure with characteristic peaks (around 1.3, 2.2, and 3.8 nm right shift compared with S3/S4 of 3.6 nm in this peak) in their pore-size-distribution curves as, in the HEMA-functionalized PDVB, the HEMA content or VBC added in ethanol could be used to tune the abundance in triple peaks of pore distribution. From Figure 5, when decreasing the overall content of HEMA (cross-linking degree in the prepared POPs increased actually), the abundance in the micropores and narrow mesopores (roughly 1–5 nm) increases. The SSA and PV witness a rapid increase from Sample 13 with SSA of 210 m^2^/g, PV of 0.244 cm^3^/g to Sample 14 with SSA of 380 m^2^/g, PV of 0.400 cm^3^/g. Another important role that the dual functional comonomers played is that this dual functional comonomer system could adjust the bulk density and morphology of prepared POPs. As seen in Table 1, the bulk density of Sample 13 had a relatively high bulk density of 0.28 cm^3^/g, which is hard to obtain using a HEMA/DVB comonomer system given no other template agent was added. We will discuss the morphology of the prepared POPs in detail later.

### 3.4. The Influence of a Template Agent on the Pore Structure of POPs

**Figure 6:**
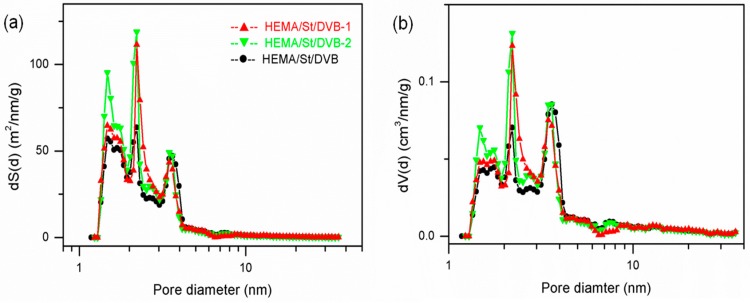
NLDFT simulation of the pore structure of POPs (Samples 8-1/8-2/9) from template methods. (**a**) Pore size distribution curves of dS(d) vs. d and (**b**) dV(d) vs. d.

We have reported in our previous research [12] that the template agent is a useful tool to tune the pore structure, bulk density, and morphology of the prepared POPs. In the prepared PDVB-based POPs with dual functional comonomers HEMA and styrene, iron oxide (II, III) aggregates of 20 nm were used as a template to tune their pore structure and morphology. As seen in Table 1, the iron oxide (II, III) template could help to improve the specific surface area and the total volume in the HEMA/St/DVB system (Samples 8-2 and 9), increasing the SSA from 183 to 215 m^2^/g and the PV from 0.296 to 0.301 cm^3^/g. Furthermore, the bulk density of the prepared poly(HEMA-co-St-co-DVB) particles increased obviously from 0.22 to 0.29 g/cm^3^. As we explained, the particle-forming mechanism using a template agent in our previous work [12], when a hydrophilic metal oxide was used as a template agent, the hydrophilic functional comonomers would adsorb on the interfaces of nanoaggregates of the iron oxide (II, III). After a free-radical initiator initialized the copolymerization on their interfaces, the poly(HEMA-co-St-co-DVB) particles were prepared around the nanoaggregates of iron oxide (II, III). As the HEMA, styrene, and DVB monomers continued to diffuse and polymerize on the surface of the metal oxide, the POP particles continuingly grew larger and finally the metal oxide or nanoaggregates were dispersed in the matrix of the prepared POPs. After acid etching, the metal oxide was removed and the final POPs were obtained. From Figure 6, we can observe an overall increase of SSA and PV of Sample 8-2 when the nanoaggregates of iron oxide (II, III) in Sample 8-1 was removed, while the pore-size-distribution curves of the three samples (8-1/8-2/9) kept similar. These results could be reasonably explained by this proposed particle-forming mechanism of the template agent. Similar results are also observed when the nanoaggregates of iron oxide (II, III) were used as a template agent in the synthesis of poly(HPMA-co-DVB) particles (see Samples 7-1/7-2/4).

### 3.5. Cross-Linking Degree and Solvent on the Pore Structure of POPs

As discussed above, the cross-linking degree of the prepared POPs could be identified as an important factor to tune the pore structure of the amorphous POPs. The solubility parameters of St and DVB (55% and 80%) are very close, and their chemical properties are also similar to each other, except their cross-linking degree produced in the POPs. When decreasing the content of cross-linking degree from DVB (80%) to St/DVB (55%) (5:4), as seen from Samples 1 and 2 (DVB only) in Figure 2 and Figure 3, Sample 1 became less porous and the characteristic peak around 2.1 nm in the pore-size distribution decreased dramatically compared to the highly cross-linked PDVB networks. 

The solvent system used was also a key factor in influencing the pore size and the pore-size distribution, the bulk density, and surface morphology of the target polymer. This factor was discussed in detail in our previous work [35]; the pore structure and morphology of the prepared polymer are highly related with the thermodynamic compatibility evaluated by the difference of the solubility parameters between the polymer and the solvent system. Typically, it is very important to match the functional comonomers/DVB system with suitable solvent(s) in the design and synthesis of potential POPs as suitable metallocene-catalyst support. 

### 3.6. IR Analysis

**Figure 7:**
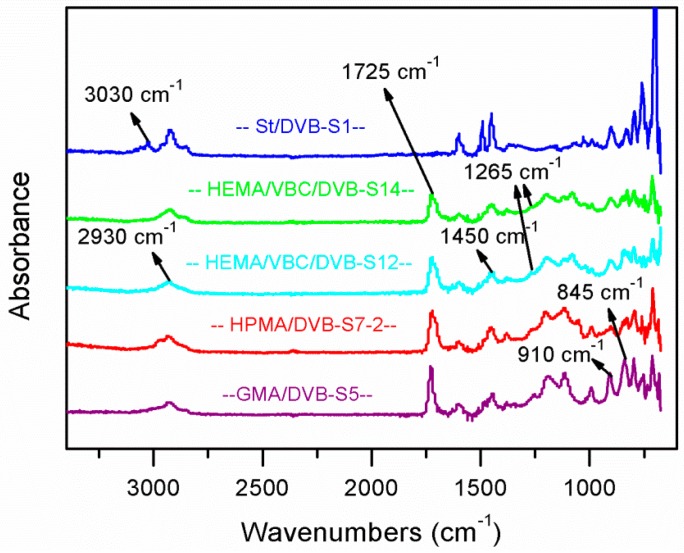
IR spectra of the prepared POPs with different functional comonomers.

The IR spectra of the five prepared functionalized PDVB samples (1, 5, 7-2, 12, and 14 with St, GMA, HPMA, HEMA/VBC, and HEMA/VBC as functional comonomers, respectively) are shown in Figure 7. We can observe that the bands in 1450 and in 2930 cm^−1^ exist in all these samples which is the inplane-bending or twist-bending mode, and the stretching vibration of $\tilde{𝛖}$(C–H) of methylene(–CH_2_–), respectively. The peak around 1725 cm^−1^ is the sketching mode of C=O in the 4 samples (5, 7-2, 12, and 14) with GMA, HPMA, and HEMA units in their own network. Generally, the bands in 1450 and in 2930 cm^−1^ can be used to calibrate relative content of the C=O functional group in these functionalized PDVB samples by peak area or peak height contrast with the peak around 1725 cm^−1^ [38,39]. Furthermore, the peak in 3030 cm^−1^ due to the stretching vibration mode of $\tilde{𝛖}$(C–H) inbenzene was observed obviously in the P(St-co-DVB) Sample 1. In the GMA-functionalized PDVB (Sample 5), except from the peak around 1725 cm^−1^, the bands around 910 and 845 cm^−1^ were also observed, which are the characteristic peaks of the asymmetic vibration of the epoxy group of GMA units, and the lack of absorbance in the range from 3600 to 3200 cm^−1^ proved that GMA units are incorporated into the P(GMA-co-DVB) network by C=C double bond, not by ring-opening polymerization of the epoxy group in GMA. In the P(HEMA-co-DVB-co-VBC) systems (Samples 12 and 14), the peak around 1265 cm^−1^ could be attributed to the characteristic absorption peak of methyl chloride linked to benzene ring, and this peak was stronger in Sample 12 than in Sample 14 due to the higher adding ratio of the VBC comonomer.

### 3.7. Bulk Density and Surface Morphology of the POPs

**Figure 8:**
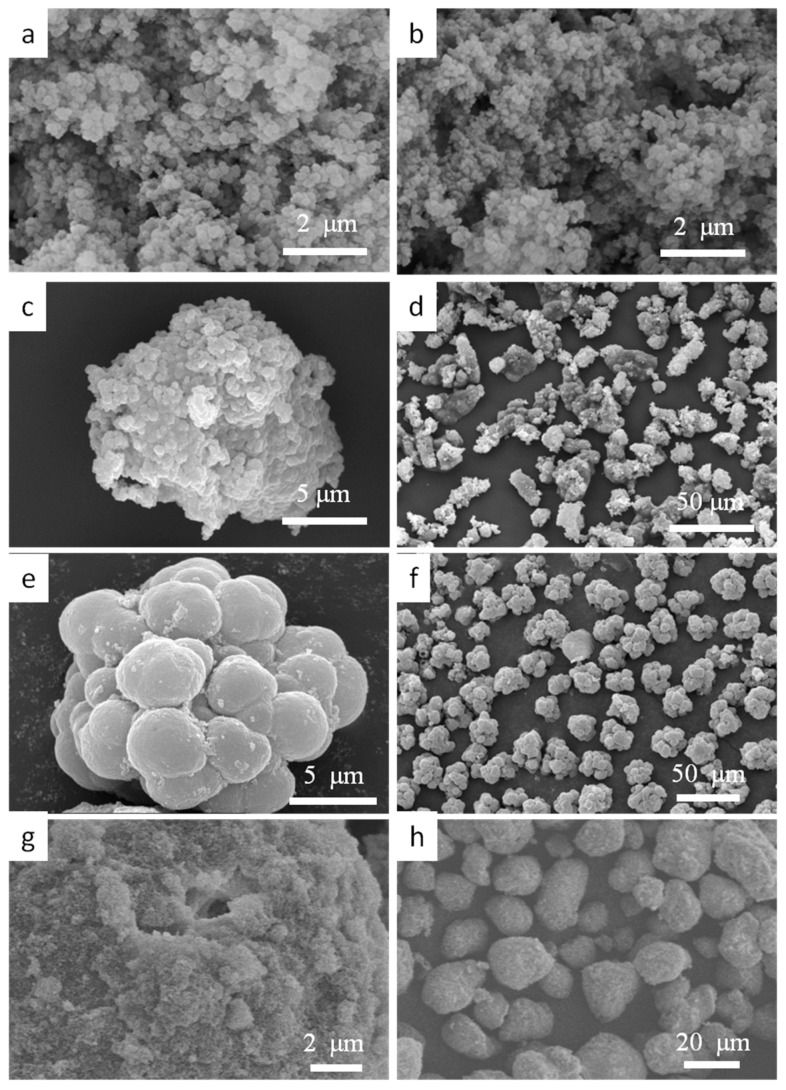
Scanning electron microscope images of functionalized POP particles. (**a**) The P(DVB) particles of Sample 2, (**b**) the P(st-co-DVB) particles of Sample 1, (**c**,**d**) the P(HEMA-co-DVB) particles of Sample 3, (**e**,**f**) the P(HEMA-co-VBC-co-DVB) particles of Sample 11, (**g**,**h**) the P(HEMA-co-VBC-co-DVB) particles of Sample 14.

By the design of the PDVB-based functionalized POPs with varied functional comonomer(s), template agent, and solvents, a wide range of bulk density of the prepared POPs (0.02–0.39 g/cm^3^) could be obtained as seen from Table 1. In the ethanol/deionized water (9:1) mixture solvents, styrene, VBC, GMA, and DVB and their own polymer networks exhibited bad thermodynamic compatibility with the solvents system, which caused the early-phase separation of the prepared POPs and low bulk density (0.02–0.08 g/cm^3^). The SEM images of representative POPs were shown in Figure 8. From Figure 8, we can see that the P(St-co-DVB) and the P(DVB) particles consist of aggregates of nano-microspheres and their low bulk density was caused by fluffy stacking of the microspheres. When HEMA and HPMA functional comonomers were incorporated into the PDVB networks, the bulk density of the P(HEMA-co-DVB) and P(HPMA-co-DVB) particles increased dramatically, from 0.02 to above 0.20 g/cm^3^, due to their good thermodynamic compatibility with the same solvents system, and the SEM image of S3 proved that the dramatic increase of bulk density was due to the compact stacking of the microspheres. Furthermore, when the HEMA functional comonomer was incorporated into the P(St-co-DVB) or P(VBC-co-DVB) networks, it could also help improve the thermodynamic compatibility with the selected solvents system; the prepared P(HEMA-co-St-co-DVB) or P(HEMA-co-VBC-co-DVB) particles obtained relatively high bulk density (0.16–0.28 g/cm^3^) by dispersion polymerization. As seen from the SEM image of Sample 14, the highly porous structure and compact stacking of the aggregates was observed due to their good thermodynamic compatibility with the chosen solvents. Samples 11 and 12 were prepared in an inhomogeneous system due to the bad solubility of VBC in the used solvents system, and higher bulk density (0.33–0.39 g/cm^3^) was obtained with a bad porous structure (Figure 8e,f) in suspension polymerization. 

### 3.8. Ethylene Polymerization of Supported Metallocene Catalysts

The metallocene-catalyst systems ((n-BuCp)_2_ZrCl_2_/MAO) were immobilized on the prepared PDVB-based POPs, then the supported (n-BuCp)_2_ZrCl_2_/MAO@POPs catalysts were evaluated for ethylene polymerization in a slurry process reactor. The supported catalysts were characterized for zirconium- and aluminium-loading content, and the results of Zr, Al/Zr (molar ratio) and ethylene polymerization were shown in Table 2. From Table 2, we can see that quite different Zr and Al/Zr values were obtained, and the Zr- and Al-loading content were highly dependent on the pore structure of the prepared POPs, when MAO can be effectively immobilized on the surface of the prepared functionalized POPs. High loading content of Zr and Al were obtained on highly porous POPs—Samples 4 and 14 with SSA of 430 and 380 m^2^/g, respectively, while low loading content Zr and Al were obtained on badly porous POPs—Samples 5 and 12 with SSA of 17.1 and 4.88 m^2^/g, respectively. The ethylene-polymerization results show that high polymerization activity and productivity were obtained on the immobilized catalysts with a high Zr loading content and Al/Zr molar ratio. Samples 4 and 14-supported catalysts obtained high polymerization of 8033 and 7152 kg PE/mol Zr h bar, respectively. Compared with Sample 4, Sample 5-supported catalysts obtained low polymerization activity and productivity due to low Al/Zr- and Zr-loading content, and the Sample 12-supported catalyst exhibited no polymerization activity due to near lack of Zr loading on its badly porous surface.

The bulk density of the obtained PE could be explained by the replicating mechanism of the polyethylene on the support in the polymerization process proposed by Fink and coworkers [40,41]. The higher the bulk density of the prepared POPs is, the higher the bulk density of the obtained PE from them can be achieved. The PE from Sample 4 (0.24 g/cm^3^) obtained the highest bulk density of 0.30 g/cm^3^, while the PE from Sample 5 (0.08 g/cm^3^) obtained the lowest bulk density of 0.11 g/cm^3^.

From the polymerization results, we can see that the support plays a significant role on the supported metallocene catalyst and the polyolefin product. Therefore, the design and synthesis also is critical to the supported catalysts and the polyolefin product, especially on how to control the pore structure and the bulk density by choosing suitable functional comonomer(s) on the PDVB backbone.

## 4. Conclusions

A feasibility study on the design and synthesis of POP supports with a tunable pore structure and high performance of ethylene polymerization was conducted by the selection of functional comonomers, template agent, and control of the cross-linking degree of their frameworks. Functional porous organic polymers with a tunable pore structure and varied particle morphology were designed and synthesized, and the pore-size distributions of POPs are generally wider than the crystalline polymers, and the micropores and the mesopores could be tuned at the same time by the dispersion polymerization method. A single functional comonomer or dual comonomers in the PDVB-based POPs played a significant role in the porous structure and particle morphology of the prepared polymers, and different N_2_ isotherms and pore structure with SSA of 10–450 m^2^/g, PV of 0.05–0.5 cm^3^/g, and bulk density with a range of 0.02–0.40 g/cm^3^ were obtained by the varied functional POPs, mainly due to their different functional groups and thermodynamic compatibility with the selected solvent system. Furthermore, the Fe_3_O_4_ nanoaggregates as a template agent could improve the porous structure and bulk density of the prepared POPs, and the highly cross-linking networks can dramatically increase the porous structure of the prepared POPs. The pore structure of the prepared POPs had a profound influence on the loading amount of the Zr and Al of the active sites, and the typically highly porous structure of the POPs would contribute the immobilization of the active species. High ethylene-polymerization activities of 8033 and 7152 kg PE/mol Zr h bar were achieved on the POPs-supported catalysts, especially when high Al/Zr ratios on the catalysts were obtained. By replicating the effect, the produced PE obtained higher bulk densities from the POPs with higher bulk densities. Therefore, it is a facile and practical approach to tailor the active sites of the metallocene catalysts and polyolefin particle morphology through the design of pore structure and surface morphology of the prepared POPs.

## Figures and Tables

**Table 1:** Characterization of poly(divinylbenzene) (PDVB)-based functional polymer from N_2_ sorption results and bulk density.

No.	Functional Comonomer (FC)	Solvent	Divinylbenzene (DVB)	FC/DVB (Molar Ratio)	Template Agent	Specific Surface Area(Multi Point BET) [m^2^/g]	Total Pore Volume [cm^3^/g]	Average Pore Diameter [nm]	Bulk Density g/cm^3^
1	St	EtOH/H_2_O = 10:1	55%	5:4	-	41.6	0.0836	8.03	0.08
2	--	EtOH/H_2_O = 9:1	80%	-	-	178	0.396	8.89	0.02
3	HEMA	EtOH/H_2_O = 9:1	80%	2:3	-	417	0.434	4.16	0.20
4	HPMA	EtOH/H_2_O = 9:1	80%	2:3	-	430	0.447	4.16	0.24
5	GMA	EtOH/H_2_O = 9:1	80%	3:5	-	17.1	0.040	9.34	0.08
6	VBC	EtOH/H_2_O =9:1	80%	0.52:1	-	50.9	0.104	8.21	0.05
7-1	HPMA	EtOH/H_2_O = 9:1	80%	2:3	Fe_3_O_4_-20 nm 3 wt %	412	0.420	4.08	0.25
7-2					-	441	0.480	4.35	0.24
8-1	HEMA/St (FC1/FC2)	EtOH/H_2_O = 9:1	80%	FC1/FC2/DVB = 1.0/0.7/1.15	Fe_3_O_4_-20 nm 3 wt %	199	0.299	6.01	0.30
8-2						215	0.301	5.59	0.29
9	HEMA/St (FC1/FC2)	EtOH/H_2_O = 9:1	80%	1.0/0.7/1.15	-	183	0.296	6.45	0.22
10	HEMA/VBC	EtOH/H_2_O = 9:1	80%	1:0.29:1.5	-	90.8	0.122	5.32	0.16
11	HEMA/VBC	EtOH/H_2_O = 9:1	80%	1:0.56:1.19 *^a^	-	15.1	0.0383	10.1	0.39
12	HEMA/VBC	ethanol	80%	1:0.32:1.19 *^a^		4.88	0.0131	10.7	0.33
13	HEMA/VBC	ethanol	80%	1:0.147:1.19	-	210	0.244	4.63	0.28
14	HEMA/VBC	ethanol	80%	1:0.17:1.5	-	380	0.400	4.21	0.22

*^a^ The reaction solution after stirring is not homogeneous system due to bad thermal compatibility. HEMA: 2-hydroxyethylmethacrylate; HPMA: 2-hydroxypropylmethacrylate; GMA: glycidyl methacrylate; VBC: vinylbenzyl chloride.

**Table 2:** Ethylene polymerization results (catalyst: (n-BuCp)_2_ZrCl_2_/MAO@POPs) *^b^.

No.	Zr (μmol/g)	Cat (mg)	Al/Zr Molar Ratio	Yield (g)	Activity Kg PE/mol Zr h bar	Productivity g PE/g cat h	Bulk Density g/mL
S4	15.6	208	272	39.1	8033	376	0.30
S5	2.3	192	179	1.45	2189	15.1	0.11
S12	0.6	184	247	-	-	-	-
S14	16.7	211	254	37.8	7152	358	0.29

*^b^ Slurry polymerization condition: 3 bar ethylene pressure in 800 mL reactor of stainless steel, 350 mL hexane, 80 °C, 2 mL (1 M in hexane) TIBA (scavenger), polymerization time: 30 min.
